# Employing Natural Control for Confounding Factors in the Hunt for the Bilingual Advantage in Attention: Evidence from School Children in Gibraltar

**DOI:** 10.5334/joc.94

**Published:** 2020-03-20

**Authors:** Chris M. Moreno-Stokoe, Markus F. Damian

**Affiliations:** 1 University of Bristol, UK

**Keywords:** Bilingualism, Executive functions, Auditory Attention, Attentional Components

## Abstract

Does being bilingual convey a benefit in ‘cognitive control’? Research on this question has been plagued by confounding geo-political factors which themselves might affect cognitive ability (e.g., Socio-Economic Status, immigration and culture). In the current study, we addressed this problem by exploring individuals of varying degrees of bilingualism from one and the same population, hence naturally controlling for confounding variables. The English/Spanish speaking population of Gibraltar share the same education, amenities, and culture on a very small landmass but vary in the degree to which they master multiple languages. We assessed the performance of 207 Gibraltarian children (9–10 yrs) on a battery of auditory attention tests and captured their degree of bilingualism via self-reported and ‘objective’ methods. We found at least ‘moderate’ evidence that measures of bilingualism cannot predict attentional ability. These results add to growing scepticism concerning the truthfulness of the claim that bilingualism conveys cognitive advantages.

## Introduction

### The history of the bilingual advantage

The scientific consensus on the effects of bilingualism on cognitive development and control has changed greatly over time. Classic research c.1920–1960 postulated that bilingualism reduces one’s IQ, delays language development, lowers self-confidence and could even cause schizophrenia (Jensen, 1962). Of course, most of these claims failed to be replicated; they were driven by anti-immigration politics of the time and used poor methodology (Darcy, 1963; Jensen, 1962; Hakuta, 1986). However, this research inspired Peal and Lambert’s (1962) landmark study which found that bilinguals were often immigrants, with less education as well as worse-paying jobs, and when matched on these factors, monolinguals and bilinguals had the same IQs. Methodological research highlighted the need for controlling for covariables and effectively ended this era of ‘bilingual handicap myths’ (Darcy, 1963; Jensen, 1962; Soto, 1997).

Subsequent research found that bilinguals would often perform better on tests of attention (Cummins, 1978; Green, 1998; Kessler & Quinn, 1987; Soto, 1997). This hypothesised bilingual advantage in attention, as opposed to an advantage in another cognitive or linguistic domain (for review see: Adesope et al., 2010), is the focus of this paper and hereon will simply be referred to as the bilingual advantage. Attention is often subdivided into an ‘Attentional Network’ of three functionally and neurologically distinct systems: executive control involves the inhibition of behaviours and resolving conflicting information, orienting directs attention towards relevant stimuli and alerting involves a receptiveness to incoming information (Fan, Worden, Guise et al., 2007; Posner & Raichle, 1994). Green (1998) argued that inhibiting the activation of one language when speaking in another trains executive control. Sometimes termed the ‘inhibition hypothesis’, according to this view bilinguals appear to have neurological changes corresponding to better executive control (Olulade, Jamal, Koo et al., 2016), and the erroneous activation of irrelevant words (e.g., from other languages) conflicts with and slows down the processes of speech and comprehension (Treccani, Argyri, Sorace & Della Sala, 2009; Spalek et al., 2014). Hilchey and Klein (2011) put forward an alternative claim, the ‘switching theory’: switching from one language to another trains one’s global ability to direct their focus of attention (orienting: Carlson, 2008; Martin-Rhee & Bialystok, 2008; Hilchey & Klein, 2011). The implications of the hypothesised bilingual advantage are substantial, for example that learning a second language could improve one’s everyday attention abilities, or even stave off attentional decline in dementia (Bialystok, Craik & Freedman, 2007).

Research c.2000–2010 appeared to support the bilingual advantage since it was reported in a variety of visual attention tests in adults and children including: Stroop and Flanker ‘conflict resolution’ tests (e.g., Bialystok, Craik & Lux, 2008; Costa, Hernandez & Sebastian-Galles, 2008; Yang, Yang, Ceci & Wang, 2005), Simon and Go/No-Go ‘inhibitory control’ tests (e.g., Bialystok, Craik & Ryan 2006; Carlson & Meltzoff, 2008), and Dimensional Change and Wisconsin Card Sort ‘task switching’ tests (e.g., Bialystok, Craik, & Ryan 2006; Carlson & Meltzoff, 2008). Furthermore, meta-analyses during this period were favourable (Adesope, Lavin, Thompson, & Ungerleider, 2010; Hilchey & Klein, 2011), especially with older adults and young children (presumably due to their attentional limitations compared to young adults), and bilingualism appeared to delay dementia attentional decline (Bialystok, Craik, Klein & Viswanathan, 2004; Bialystok, Craik & Freedman, 2007; Chertkow, Whitehead, Phillips et al., 2010). In this period, research broadly supported Green’s theory and the consensus was that the bilingual advantage hypothesis had received overwhelming support (Adesope, Lavin, Thompson & Ungerleider, 2010).

However, since 2010 this consensus has been challenged, in light of recent reviews which suggest that the bilingual advantage is not as strong as it is claimed to be, or may not even exist at all (e.g., Chen, Zhou, Uchikoshi & Bunge, 2014; DeBruin, Treccani & Della Salla, 2015; Duñabeitia & Carreiras, 2015; Paap & Greenberg, 2013; Paap, Johnson & Sawi, 2016). Paap, Johnston and Swami’s (2015) meta-analysis suggested that 80% of post-2011 research found no bilingual advantage. Meta-analyses combining modern and pre-2011 papers also supported the null (Anton, Duñabeitia, Estévez et al., 2014; DeBruin, Treccani & Della Sala, 2015; Duñabeitia & Carreiras, 2015; Paap & Greenberg, 2013), in stark contrast with studies published pre-2011 which often found an advantage (Adesope, Lavin, Thompson & Ungerleider, 2010; Hilchey & Klein, 2011). As a consequence, whether bilingualism affords cognitive advantages is currently unresolved and remains highly controversial.

### Poor methods in researching the bilingual advantage in attention

Research on the bilingual advantage has suffered from poor methodology, small sample sizes and flawed measures which show poor convergent validity (Paap, Johnson & Sawi, 2015), as well as a publication bias which resulted in studies supporting the advantage being more likely to be published (DeBruin, Trecanni & Della Salla, 2015). Further, results appear dependent on whether studies use auditory or visual stimuli, verbal or non-verbal stimuli, as well as the test language (Barac & Bialystok, 2012; Bak, Vega-Mendoza & Sorace, 2014; Foy & Mann, 2014; Roebuck, Freigang & Barry, 2016; Spagna, Mackie & Fan, 2015; Stewart & Armitay, 2015). For example, a participant’s response to verbal stimuli are affected by, and therefore confounded by, language ability, whereas this is not true for non-verbal stimuli. On the other hand, visually presented stimuli involve visual cognitive processes whereas auditory stimuli do not and yet these are less utilised (Anton et al., 2014). The language in which stimuli were presented in also been found to affect results on cognitive tests, arguably due to the differences in how languages sound (Phonology: Prior & Gollan, 2011; Gollan et al., 2014; Declerck & Phillip, 2015; Phillip & Koch, 2016). Therefore stimuli and presentation modality selection as well as test language are greatly important. Paap, Johnson and Sawi (2015, 2016) highlight the lack of internal consistency between different measures of attention even though they are supposed to measure similar constructs (e.g., between Simon and Flanker tasks).

Another key issue is the lack of consistency in the definition of ‘bilingualism’: bilinguals are not all the same and vary on many variables including geo-political (e.g., country of domicile, collectivist culture, socio-economic status and immigration status) and linguistic factors (frequency, and balance, of bilingual language usage; language proficiency; age of learning languages; as well as bilingual experiences and micro-cultures; Adesope, Lavin, Thompson, & Ungerleider, 2010; Bialystok, 2001; Carlson and Meltzoff, 2008; Duñabeitia & Carreiras, 2015; Hamers & Blanc, 2000; Paap, Johnson & Sawi, 2016; Tao et al., 2011; Tran, Arredondo & Yoshida, 2015).

Matching bilinguals with a control monolingual population has proven difficult as well. Bilinguals often differ from monolinguals on factors such as education, job title and immigrant status (Paap, Johnson & Sawi, 2015; Peal & Lambert, 1962) that in combination determine Socio-Economic Status (SES). SES has been linked to executive control ability, perhaps because SES affects the provision of emotional and academic resources during childhood (Conger, Ge, Elder et al., 1994; Conger, Patterson & Ge, 1995; Linver, Brooks-Gunn & Kohen, 2002). Studies in which bilinguals and monolinguals were explicitly matched on SES often found no bilingual advantage (Farah & Noble, 2005; Noble, Norman & Farah, 2005; Morton & Harper, 2007; Paap, Johnson & Sawi, 2015). Furthermore, collectivist culture has been hypothesised to encourage a parenting style which teaches children to be more receptive to incoming information and therefore may improve one component of attention (‘alerting’: Tran, Arredondo & Yoshida, 2015). Lastly, research has identified many additional variables which have been theoretically or practically linked to attention and may confound with bilingualism in any given study or population: genetics, gender, education, music training, computer usage and video gaming, exercise and sport, family values, cultural values and social interaction (Adesope, Lavin, Thompson & Ungerleider, 2010; Bialystok & DePape, 2009; Bialystok, Craik & Ryan, 2006; Paap, Johnson & Sawi, 2015; Tran, Arredondo & Yoshida, 2015). These factors combine to form complex dynamical systems in which factors affect individuals differentially, which makes controlling for them very difficult (Hilchey & Klein, 2011). Hence, whether or not these variables confound studies in which mono- and bilinguals are compared is at present unclear and with the exception of SES and immigration, has not received sufficient attention in research.

### The present study

In the study reported below, we explored whether various confounders might have contributed to previous findings of bilingual advantages. A partnership was made with HM Government of Gibraltar Department of Education to recruit a large sample of middle school children. Gibraltar is very small (6.7 km^2^) and so everyone in the population shares the same health, leisure, sport, education and commercial facilities which provide a natural control for many confounding variables. At the same time, due to history, politics, and proximity to Spain, the Gibraltarian population are diverse in language background and range from almost entirely monolingual (mostly in English) to fully bilingual in English and Spanish language. It is therefore possible to compare monolinguals and bilinguals within this same population. The present study utilised this natural control as well as conducting correlation analysis to investigate the degree to which three major factors (SES, culture, immigration) correlate with bilingualism and attention. To our knowledge, this is the first natural control in the literature on the bilingual advantage in attention.

For our participants, we captured bilingualism as a continuous variable (for discussion see: Luk & Bialystok, 2013; von Bastian, Souza & Gade, 2016; Incera & McLennan, 2017). A receptive vocabulary test loosely adapted from the Peabody picture-vocabulary test (Dunn & Dunn, 2007) was used to separately quantify individuals’ English and Spanish vocabularies. In addition, we administered a subjective language behaviour questionnaire which captured bilingual behaviour (e.g., Language and Social Background Questionnaire: Luk et al., 2013). Children’s attentional abilities were measured via the Auditory Attentional Network Test (aANT: Roberts, Summerfield & Hall, 2006) and the Test of Attention in Listening (TAIL: Zhang, Barry, Moore & Amitay, 2012). These allowed us to identify three components of attention (conflict resolution; orienting; alerting) as well as to measure overall response speed and accuracy. The non-verbal auditory stimuli used in these tasks were chosen to separate attention from comprehension ability (Anton et al., 2014). The analysis centred on whether one or more of these components of cognitive control could be predicted from individuals’ language background. Hence, overall the objective of this research was to investigate the validity of poor-confound-control criticisms with the hypothesis that: No bilingual advantage in attention will be found when employing natural covariable control.

## Method

### Participants

207 Year 5 schoolchildren were recruited from four middle schools in Gibraltar (male *n* = 108, female *n* = 99; age range = 9–10 yrs, mean age = 9 yrs, 1 mths). Due to practical constraints, not all children completed all components of assessment (see below for specific information regarding sample size on each task).

### Measures and materials

Child participants were subject to a computer test and a questionnaire which measured bilingualism; parents completed an additional questionnaire to measure other factors (SES, culture, immigration). Children also completed two computer tests measuring facets of attentional abilities. All computer-based tasks were administered using DMDX (Version 5.1.3.4; Forster & Forster, 2003).

#### 1. Measures of Bilingualism

To measure the degree of ‘bilingualism’, participants were tested in their proficiency and usage of the English and Spanish languages. Bilingualism was measured on a continuous scale (rather than comparing bilingual to monolinguals categorically) in order to accurately capture variation between bilinguals (Chen, Zhou, Uchikoshi & Bunge, 2014; Hurtado, Gruter, Marchman & Fernald, 2014; Incera & McLennan, 2017).

##### A) Bilingual Language Vocabulary test (BilVoc)

**Figure 1:**
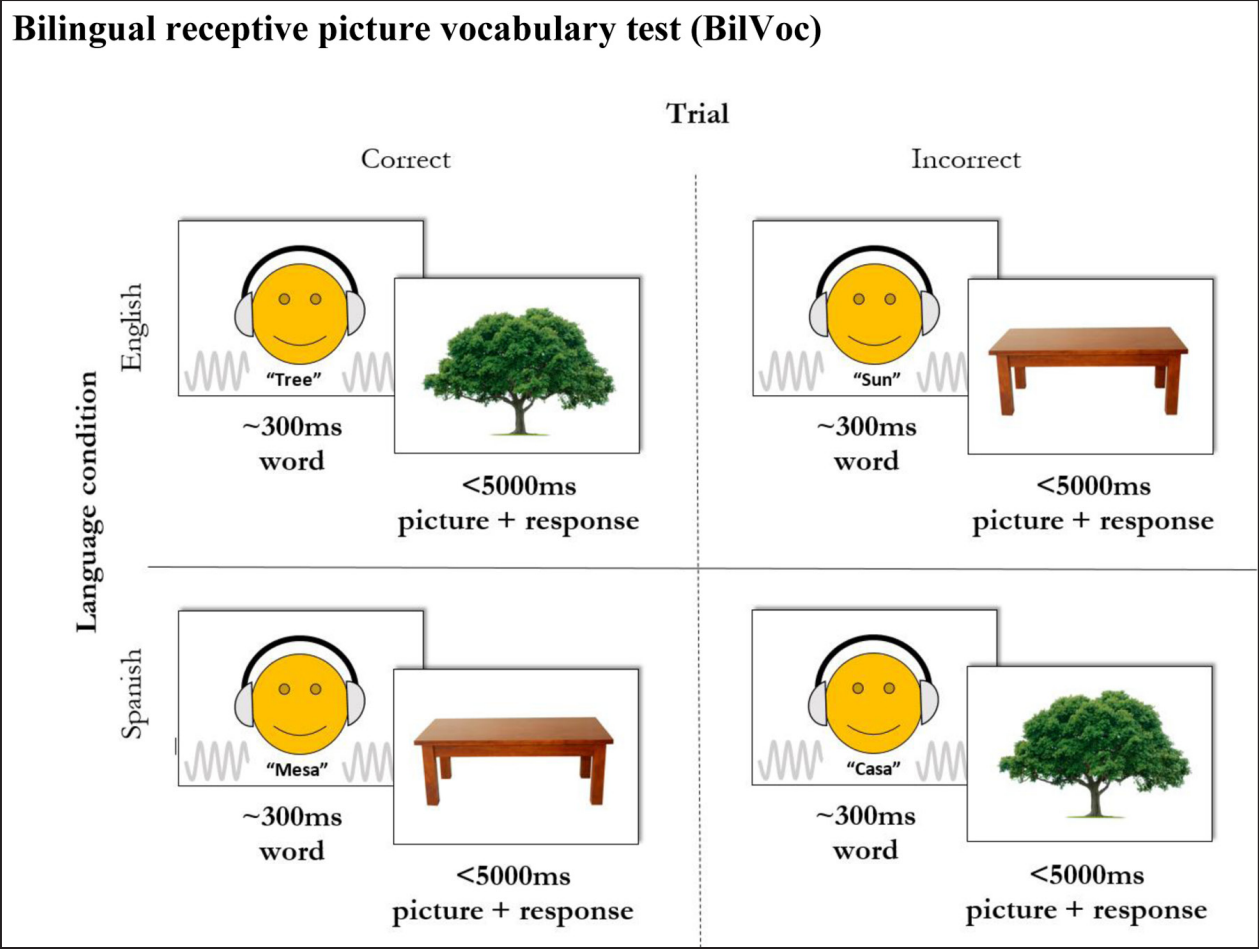
Schematic illustration of the bilingual picture vocabulary test (BilVoc).

A language proficiency test, the Bilingual Language Vocabulary test (BilVoc), was developed based on the Peabody-IV Picture Vocabulary Task which measures language proficiency by assessing receptive vocabulary (Dunn & Dunn, 2007). The BilVoc displayed an image of an object along with a computerised, emotionally neutral, Text-To-Speech pronunciation of a noun. Participants were tasked with indicating whether or not this noun accurately described the object in the picture, and speed and accuracy of the response were measured on each trial. Figure 1 shows a schematic illustration of this task.

Images averaged 500 × 400 px, sourced from www.google.images.com, and were displayed in the centre of the screen. English and Spanish versions of the BilVoc were produced. The MRC Psycholinguistic Database (Wilson, 1987) was used to randomly select 80 nouns from the English corpus. Nouns were stratified by age of acquisition, with 20 words randomly selected from each of four age groups: 0–2yrs, 2–7yrs, 7–13yrs and 13+yrs (MRC age parameters: 100, 100–300, 300–500 & 500–700). This range was chosen to challenge children and to avoid ceiling effects on performance. Nouns with low imageability, such as ‘age’, were excluded. Translation equivalents of the English materials were used for the Spanish portion of the test. Age of acquisition values of the Spanish materials (derived from Alonso, Fernandez & Díez, 2015) were comparable to those of the English materials. Participants undertook 160 trials randomly arranged into 2 blocks (one block of 80 English words and one block of 80 Spanish words).

##### B) Bilingualism questionnaire (BilQ)

A novel questionnaire, the subjective bilingualism questionnaire (BilQ), was developed to assess the language usage behaviours in children. The BilQ involved indicating the degree to which one would choose to speak either English, Spanish or the local dialect combining both (Llanito) in a given situation. Questions were adapted from adult language usage questionnaires, for example “At home, I speak… English/Spanish/Both” and “I watch TV in… English/Spanish/Both” (Language Experience and Proficiency Questionnaire: Marian, Blumenfield & Kaushanskaya, 2007; Language and Social Background Questionnaire, e.g., Luk et al., 2013). Compared to adult questionnaires this questionnaire is shorter, uses simpler language and replaces 0–100% scales with relatable and imaginable situations for children. The questions are laid out in Table 1.

**Table 1:** Questions asked in the BilQ to assess language usage. For questions with sub-questions, participants were required to answer again separately for each language.

#	Questions	*Sub-question*	Possible responses

1e	I speak:	*English…*	1. Never
1s		*Spanish…*	2. A few times a month
			3. Every day
			4. Always
2e	If someone speaks English and Spanish, what language do you speak to them in?	*English…*	1. Never
2s		*Spanish…*	2. Sometimes
			3. Most of the time
			4. Always
3e	I started learning English:		1. At school
3s	I started learning Spanish:		2. Years before school
			3. I’ve always known it
4	I talk to friends in:		1. English
5	I talk to family in:		2. Spanish
6	I watch television in:		3. Both

##### C) Parental questionnaire

To capture the effect of potential confounding variables, a questionnaire was produced which asked parents for their *SES*: the highest level of education (i.e., college) and income bracket (based on Gibraltarian Employment Survey Report: HM Gov. Gibraltar, 2014); *individualist culture*: the birthplace of family members up to and including grand-parents (Geert-Hofstede individualist culture scores for each member’s country of birth were calculated and the average was taken and high scores indicate a strong individualist tendency: Geert-Hofstede, 2017); and *immigrant status*: the child’s status of residence in Gibraltar.

#### 2. Attention tasks

Three components of the attentional network were tested: Conflict resolution (executive control), orienting and alerting. Existing tests were adapted to identify these three components using the same non-verbal, auditory stimuli, as outlined below. A battery of attention-related tests (Attentional Network Test: Fan et al., 2002) was adopted to overcome previous issues of convergent validity undermining the reliability of results. Non-verbal stimuli were adopted because their interpretation is not related to linguistic ability, a potential confound between bilingualism and verbal performance (Anton et al., 2014). The Test of Attention in Listening (TAIL: Zhang, Barry, Moore & Amitay, 2012) and the Auditory Attention Network Test (aANT: Roberts, Summerfield & Hall, 2006) were adapted for our purposes. We created an auditory, non-verbal Attentional Network Test for 9–10 year olds, which we refer to here simply as the ‘ANT2’ for children. The ANT2 is a frequency discrimination task in which participants determine whether two auditory tones are the same or different in pitch. Eight perceptually distinguishable auditory tone stimuli were generated in Audacity (Version 2.1.0; Audacity Team, 2015) at least 2.1 Equivalent Rectangular Bandwidths apart and lasting 100ms (frequencies: 500 Hz, 685 Hz, 916 Hz, 1207 Hz, 1571 Hz, 2027 Hz, 2599 Hz & 3316 Hz). Stimuli were numerous and similar enough to require attentional focus to distinguish (Zhang, Barry, Moore & Amitay, 2012).

##### A) ANT2: Conflict and Orienting trials (ANT2:C&O)

**Figure 2:**
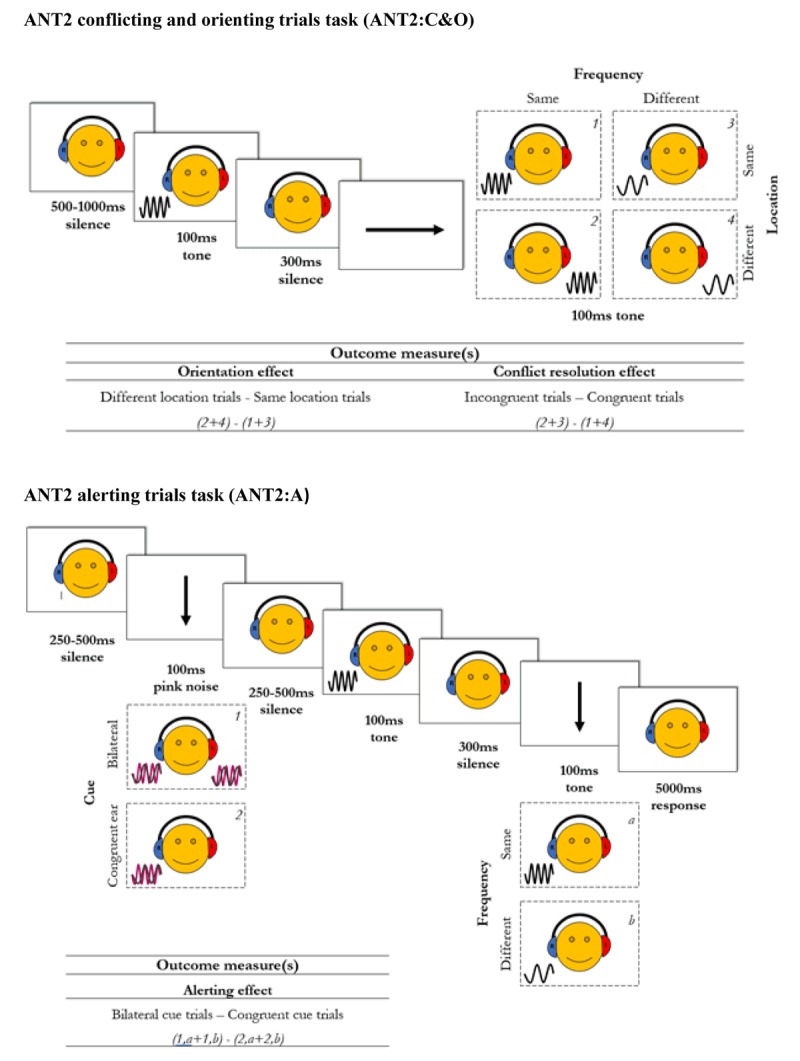
Schematic illustration of the ANT2 conflict and orienting trials (ANT2:C&O) in top panel, and of the ANT2 alerting trials (ANT2:A) in bottom panel. In the ANT2:C&O, response time was measured following the second tone (up to 5000ms).

In the first phase of the ANT2 the TAIL was adapted to provide conflict and orienting measures. These trials varied the location of the tone (i.e., left or right ear) which was irrelevant to the task but produced conflict: It is easier to respond when stimuli are the same in both properties (location and pitch) and more difficult when they conflict (different location but same pitch). Individuals with better conflict resolution (executive control) ability would be less affected by conflict. Figure 2 (top panel) shows a schematic illustration of the task. The *orienting* component of this task was measured by comparing performance on trials where children had to orient attention from one ear to the other to performance on trials where they did not (i.e., both tones in same ear). The *conflict resolution* component was computed by comparing performance on trials with agreement (i.e., same location and pitch) to trials with conflicting answers (i.e., different location but same pitch). Participants undertook 172 trials randomly arranged in four blocks of 40 with 12 practice trials (40 trials per condition).

##### B) ANT2: Alerting trials (ANT2:A)

In the second phase of the ANT2 the aANT was adapted to provide an alerting measure. These trials did not vary location but instead introduced an auditory cue (100ms of static noise) which was played in the ear where two tones were about to be administered. Individuals with better alerting abilities would be more receptive to processing the cue and therefore would benefit most from it. Figure 2 (bottom panel) illustrates this task. The *alerting* effect was computed by comparing trials where this cue was administered to the correct ear and when it was administered bilaterally as a neutral control. Participants undertook 88 trials (40 per condition) randomly arranged in 2 blocks, preceded by four practice trials.

Two accommodations were made to adapt these tests for children in order to avoid discomfort: frequencies were selected which were slightly lower than the TAIL used with adults (476–6188 Hz), and pink noise was used which is less harsh than the white noise used in the aANT.

#### Procedure

The Parental Questionnaires were given to the parents along with consent forms. Participants were tested during a scheduled IT class in their school’s computer laboratory where they were instructed to sit at a computer and to wear headphones. They were first tested on the computer tests in a counter-balanced order (BilVoc, ANT2:C&O, ANT2:A) and afterwards they were instructed how to fill in the BilQ by hand. The computer tests required a response with either the left or right shift keys on the computer keyboard. It was explained, and visibly labelled, that the left shift key (labelled green) was to be used in response to matching tone frequencies in the attention tests and to correct nouns in the BilVoc. The right shift key (labelled red) was used in response to non-matching tones or nouns. Participants were given 5 seconds in which to respond to each trial before the test moved on to the next trial. Testing sessions lasted approximately an hour.

## Results

For the results described below, we report frequentist statistics, as well as Bayes factors (*BF*s). Unless otherwise noted, we computed *BF_10_* (evidence for *H_1_* relative to *H_0_*) because intuitively these correspond to the “strength of evidence” for a given pattern (the higher the *BF*, the stronger the evidence for *H_1_*). A convention is that Bayes Factors larger than 3 indicate “moderate” evidence for the corresponding (positive or null) hypothesis, and Bayes Factors larger than 10 constitute “strong” evidence (e.g., Lee & Wagenmakers, 2014).

### 1. Measures of bilingualism

#### A) BilVoc

**Figure 3:**
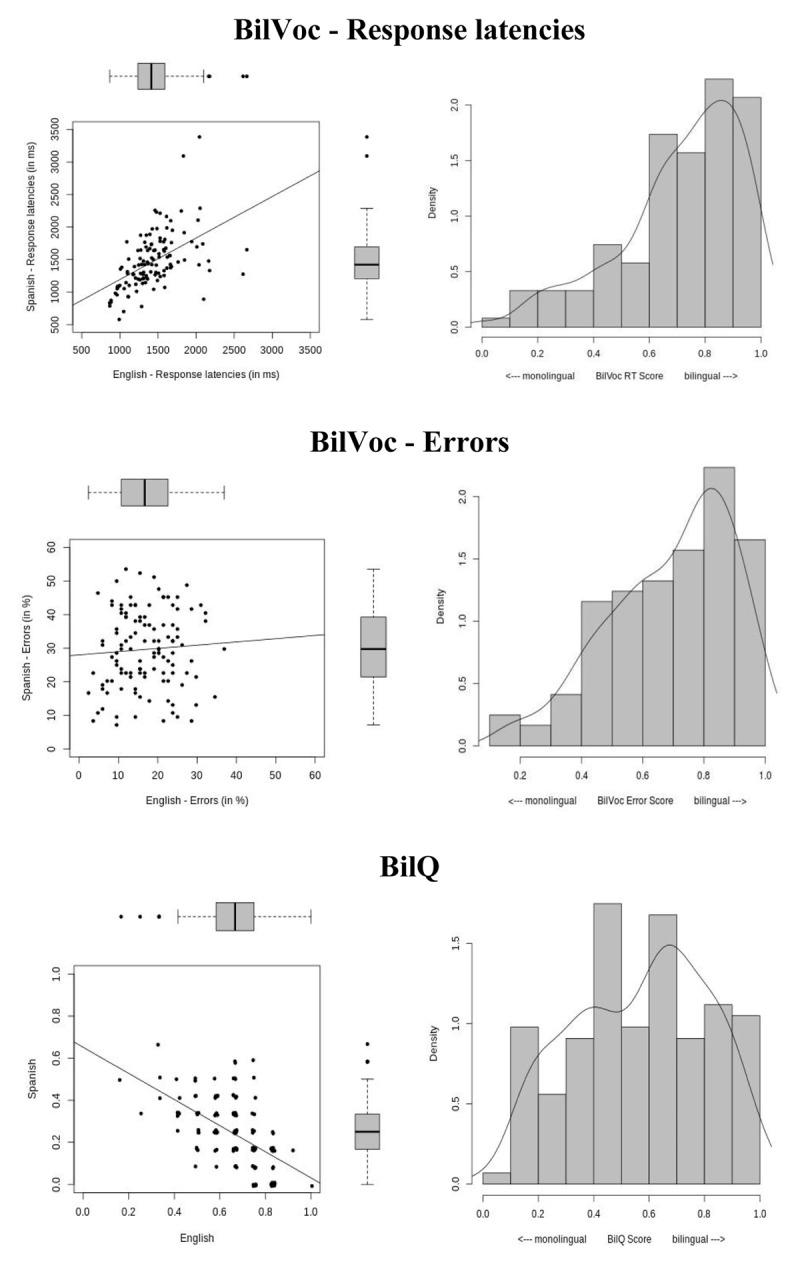
Top panel: BilVoc performance – Response latencies. Left panel: average latencies in English and Spanish plotted against each other. Right panel: density histogram of corresponding BilVoc bilingualism scores. Middle panel: BilVoc performance – error percentages. Left panel: average error scores in English and Spanish plotted against each other. Right panel: density histogram of corresponding BilVoc bilingualism scores. Bottom panel: BilQ results. Left panel: average scores on the BilQ (0–1) in English and Spanish plotted against each other. Right panel: density histogram of corresponding BilQ bilingualism scores. In all plots, points represent individual participants and have been slightly jittered to avoid overplotting.

Out of the 141 children who took the BilVoc, results from 20 children were eliminated because data loss (combined timeouts and errors) was ≥40%. Latencies from trials with errors, as well as latencies above and below 2.5 *SD* from a participant’s mean, were excluded. English response latencies (*M* = 1,346 ms, *SD* = 311) were slightly faster than Spanish latencies (*M* = 1,376 ms, *SD* = 384), but the difference was not significant, *F*(1, 120) = 1.01, *p* = .32, *BF_01_* = 0.22. Accuracy on the English portion of the BilVoc (*M* = 83.0%, *SD* = 7.4) was significantly higher than on the Spanish portion (*M* = 71.3%, *SD* = 11.4), *F*(1, 120) = 109.93, *p* < .001, *BF_10_* > 1000. The better performance in English than in Spanish was expected since the Gibraltarian population identifies more with the United Kingdom and on the whole speaks slightly more English. Figure 3 (top left panel) displays individual average latencies in English against Spanish. As indicated by the trend line, there is a substantial correlation in latencies between the two languages, *r* = .51, *p* < .001, *BF_10_* > 1000: children who are on average fast in one language also tend to be fast in the other. This association most likely simply reflects individual processing speed which is largely independent of relative skill in a given language. This could also reflect differential maturity, or language skills.

From these results, we computed a bilingualism score which reflected the relative strength of performance in the two languages. For latencies, we had to take into account the substantial differences in overall response speed, which complicate the interpretation of relative differences in average speed between the two languages. Hence, RTs were *z*-transformed separately for each participant. We then subtracted from one the absolute differences between each person’s average transformed RT in each language, and scaled the values between zero and one, with small values indicating substantial differences between RTs in the two languages, and high values indicating largely balanced RT levels across languages. A corresponding histogram is shown in Figure 3 (top right panel), exhibiting the expected right skew given that participants occupied a bilingual environment.

Figure 3 (middle left panel) displays individual average error rates in English against Spanish. Contrary to the latencies, there was no association between the two measures, *r* = .06, *p* = .493, *BF_10_* = 0.26. We computed a bilingualism error score which took into account the chance level of 50% in the task. We took the negative absolute difference between error scores in both languages, multiplied it by two, added 100, and finally divided by 100. This rendered a score between 0 and 1, with 0 indicating monolingualism (e.g., 0% errors in one language, but chance performance of 50% in another: (–2*abs(0–50)) + 100/100 = 0) and 1 indicating perfect bilingualism (e.g., 20% errors in both languages: (–2*abs(20–20)) + 100/100 = 1). A corresponding histogram is shown in the middle right panel, showing a rightward skew similar to the one obtained for latencies.

In summary, the BilVoc task exhibited the anticipated degree of difficulty for participants, with a good range of average latencies in both languages and substantial but not excessive error rates. Bilingualism scores, although reflecting the largely bilingual environment which our participants inhabited, exhibited a substantial range in values, from quasi-monolinguals to nearly perfectly balanced bilinguals.

#### B) BilQ

Performance on the BilQ was scored as an integer on a range from 5–17 separately for each language, with 5 indicating little or no usage and 17 indicating maximum usage (see online supplementary information, Appendix S1 for scoring). Scores for each language were then re-scaled to range from zero to one. Figure 3 (bottom left panel) displays English and Spanish scores against each other for the 143 participants who completed the measure. Unsurprisingly, participants were stronger in English (*M* = 0.66, *SD* = 0.14) than in Spanish (*M* = 0.14, *SD* = 0.16), with a negative correlation between the scores, *r* = –.55, *p* < .001, *BF_10_* > 1000. To form a measure of bilingualism, we subtracted from one the absolute difference of the English and Spanish scores for each participant, which resulted in a score ranging from zero (monolingual) to 1 (perfectly bilingual). Figure 3 (bottom right panel) displays a density histogram of the scores, demonstrating that the participants of the current study covered the entire range of possible scores.

#### C) Parental questionnaire

The parental questionnaire quantified SES by weighting income and education 50:50 on Likert scales to produce a measure from 6–12 (*M* = 8.51, SD = 2.64). Individualist culture, as measured on 1–100 Geert-Hofstede scores, showed a wide range of 14–89 (*M* = 69.01, *SD* = 12.46). For subsequent analysis (see below) both measures were re-scaled to a range of 0–1. Immigrant status was assigned to 41 children who were not born in Gibraltar.

Table 2 presents a correlation matrix which includes the three measures of bilingualism (bilingualism score derived from a) BilVoc RT latencies; b) BilVoc errors; c) BilQ questionnaire; recall that these range from zero to one, with zero indicating monolingual and one indicating balanced bilingual), alongside with the three demographic indicators, namely a) Individualist culture index; b) an estimate of SES; and c) Immigration status. We report Pearson correlation coefficients (*r*) as well as corresponding *p* values. Additionally, we computed Bayes factors for each correlation using *JASP* (2018), separately listing *BF_01_* (evidence for *H_0_* relative to *H_1_*) as well as *BF_10_* (evidence for *H_1_* relative to *H_0_*). These do of course convey identical information as one is the reciprocal of the other.

**Table 2:** Correlation matrix for disparity between English and Spanish language measures (BilVoc response times; BilVoc errors; and BilQ) and demographic factors (individualist culture; SES; and immigrant status).

		Bilingualism score for BilVoc RTs	Bilingualism score for BilVoc Errors	Bilingualism score for BilQ	Individualist culture	SES

**Bilingualism score**	*r*	–.206				
**for BilVoc Errors**	*p*	.024				
	*BF_01_*	0.70				
	*BF_10_*	1.42				
**Bilingualism score**	*r*	–.249	.377			
**for BilQ**	*p*	.011	<.001			
	*BF_01_*	0.33	0.01			
	*BF_10_*	3.07	**285.09**			
**Individualist**	*r*	.065	–.284	–.124		
**culture**	*p*	.502	.003	.159		
	*BF_01_*	**6.66**	0.11	**3.43**		
	*BF_10_*	0.15	**9.44**	0.29		
**SES**	*r*	–.133	–.126	–.016	–.018	
	*p*	.165	.191	.857	.810	
	*BF_01_*	**3.24**	**3.60**	**9.04**	**10.36**	
	*BF_10_*	0.31	0.28	0.11	0.10	
**Immigrant**	*r*	.085	–.017	–.170	–.131	.008
**status**	*p*	.382	.865	.052	.081	.912
	*BF_01_*	**5.72**	**8.23**	1.41	2.35	**10.71**
	*BF_10_*	0.18	0.12	0.71	0.43	0.093

*Note*: Bayes Factor (BF) values which suggest at least ‘moderate’ evidence (*BF* ≥ 3) are shown in bold.

As can be seen, the strongest evidence is for a positive relation between bilingualism as indicated by the BilQ, and the BilVoc errors. This pattern makes sense: individuals who on the BilQ indicated that they are largely bilingual tend to have a “balanced” error profile (i.e., make similar amounts of errors) on the BilVoc picture-word test. Interestingly, however, the correlations between the three measures of bilingualism, and the three demographic indicators (individualist culture; SES; and immigrant status) for the most part offer moderate evidence for the null hypothesis (seven out of nine correlations show this pattern). This confirms that in our sample of Gibraltarian school children, the confounds which normally make a comparison between mono- and bilingual individuals difficult (see Introduction) are irrelevant: at least in this group, the degree to which a child speaks more than one language is seemingly independent of variables such as SES and immigrant status. One notable exception is a substantial negative correlation between the individualist culture index and the bilingualism score as revealed in the BilVoc errors (*BF_10_* = 9.44): the higher a participant’s score of individualist culture, the less balanced their error profile was on the BilVoc test. This is not surprising considering families with more individualist culture are often English in ancestry, and therefore the child can be expected to speak better English than Spanish (United Kingdom and Spain have respective Geert-Hofstede individualist culture scores of 89 and 51 out of 100).

These results add to mounting evidence that it is difficult to truly measure bilingualism, and that one test cannot be relied upon. Internal consistency analysis was conducted to test the reliability of the behavioural bilingual language questionnaire (BilQ); the English items received a low Cronbach’s alpha (*α* = .54) whereas the Spanish items received an acceptable alpha (*α* = .75) suggesting that items in the BilQ were more convergent in assessing Spanish language usage than English.

### 2. Attention tasks

#### A) ANT2: Conflict and Orienting trials

**Figure 4:**
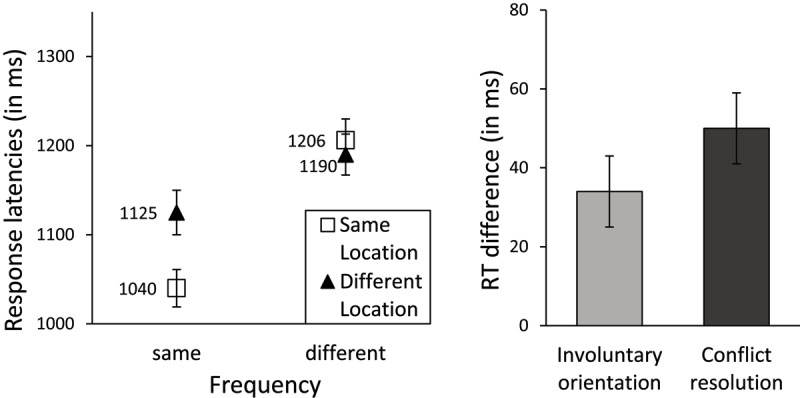
Performance on the ANT2 conflict and orienting trials (ANT2:C&O) task. Response latencies (in ms; left panel) varied by Frequency and Location; Response time differences corresponding to orientation and conflict resolution (in ms; right panel). Error bars indicate standard error of the means.

Out of the 155 children who took this task, results from 48 were eliminated because data loss (combined time outs and errors) was ≥40%. For data from the remaining 107 participants, latencies from trials with errors (19.4%), latencies faster than 300 ms (0.4%), and latencies above or below 2.5 *SD* from a participant’s mean (2.4%) were deleted. The latency results are shown in Figure 4. The cross-over interaction between frequency and location reported by Zhang et al. (2013) is clearly visible. A repeated-measures Analysis of Variance (ANOVA) revealed an effect of frequency, *F*(1, 106) = 75.25, *p* < .001, *BF_10_* > 1000, an effect of location, *F*(1, 106) = 13.40, *p* < .001, *BF_10_* = 3.3, and an interaction between frequency and location, *F*(1, 106) = 30.20, *p* < .001, *BF_10_* > 1000. Orientation and conflict resolution scores were computed in accordance with the equation in Figure 2 and are shown in the right panel of Figure 4. The orientation effect (34 ms) differed significantly from zero, *t*(106) = 3.66, *p* < .001, *BF_10_* = 50.7, and so did the conflict resolution score (50 ms), *t(*106) = 5.50, *p* < .001, *BF_10_* > 1000.

**Figure 5:**
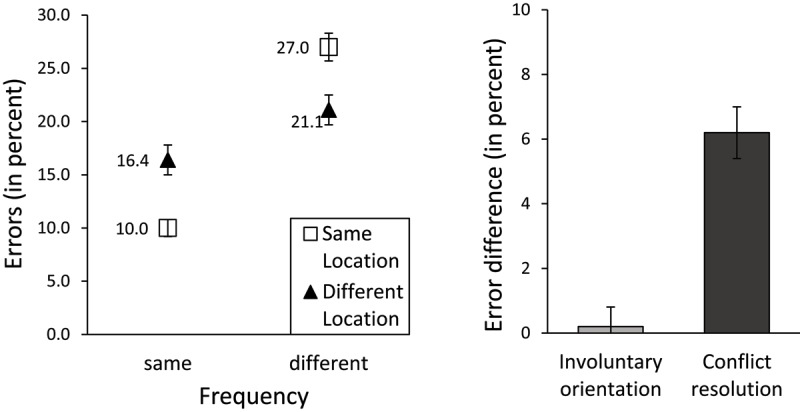
Performance on the ANT2 conflict and orienting trials (ANT2:C&O) task. Error rates (in percent; left panel) varied by Frequency and Location; Error rate differences corresponding to orientation and conflict resolution (in percent; right panel). Error bars indicate standard error of the means.

Error rates were calculated in a parallel manner and are shown in Figure 5. Error rates again show the characteristic cross-over interaction between frequency and location. An ANOVA showed an effect of frequency, *F*(1, 106) = 54.71, *p* < .001, *BF_10_* > 1000 no effect of location, *F*(1, 106) = 0.15, *p* = .70, *BF_10_* = 0.1, and a frequency by location interaction, *F*(1, 106) = 58.87, *p* < .001, *BF_10_* > 1000. Orientation and conflict resolution scores, displayed in the right panel of Figure 5, showed no orientation effect (0.2%), *t*(106) = 0.39, *p* = 0.699, *BF_10_* = 0.1, but a significant conflict resolution effect (6.2%), *t*(106) = 7.67, *p* < .001, *BF_10_* > 1000.

In summary, both latencies and error rates replicated the characteristic pattern of results (an interaction between frequency and location) reported by Zhang et al. (2013). Contrary to their results, our findings showed a frequency effect on latencies, with “same frequency” responses carried out faster than “different frequency” responses. Furthermore, we replicated the significant orientation and conflict resolution effects on latencies, but contrary to Zhang et al.’s findings, only the conflict resolution effect was significant in the error analysis.

#### B) ANT2: Alerting trials

Out of the 139 children who took this task, results from 61 were eliminated due to excessive data loss (combined errors and timeouts ≥40%). For the remaining 78 participants, latencies from trials with errors (20.4%), latencies faster than 300 ms (0.4%) and latencies above or below 2.5 *SD* from a participant’s mean (2.3%) were deleted. Latencies showed a mean of 1,177 ms (*SD* = 292) for the “congruent” condition, and one of 1,173 ms (*SD* = 310) for the “incongruent” condition. The resulting alerting effect of 4 ms did not differ significantly from zero, *t*(77) = 0.20, *p* = .843, *BF_10_* = 0.13. Error rates showed an average of 18.5% (*SD* = 9.9) for the “congruent” condition, and of 21.1% (*SD* = 10.5) for the “incongruent” condition. The alerting effect of 2.6% differed significantly from zero, *t*(77) = 2.76, *p* =.007, *BF_10_* = 4.2.

In summary, the alerting effect reported by Roberts et al. (2006) emerged significantly in the error rates but was not found in the latencies of our data.

### Assessing the bilingual advantage in attention

In a final and crucial analysis, we explored the extent to which the degree of attentional control, as manifested in “global measures” (overall RT latencies and error rate on the ANT2) and/or in “component measures” (conflict, orienting, and alerting, as measured in latencies and errors), can be predicted from the three measures of bilingualism (bilingualism scores derived from the BilVoc response latencies and errors, as well as from the BilQ self-assessments). Table 3 shows a correlation matrix between these variables; as in Table 2, we computed not only conventional statistics but also Bayes factors which allowed us to quantify the evidence for the positive as well as the null hypothesis. The only instance of a positive finding was a positive correlation (*r* = .32) between bilingualism as indexed by the BilVoc latencies, and overall RTs on the ANT2 conflicting and orienting trials *(BF_10_* = 11.4). This implies that participants who on the BilVoc exhibited relatively “balanced” average response times in English and Spanish (hence indicating a high degree of bilingualism) tended to be *slower* on the global RT measure of the ANT2 conflicting and orienting trials. Note that this pattern contradicts the previously hypothesised possibility that bilingualism results in an overall efficiency advantage (which would have predicted a negative correlation). Other than this particular finding, the overall pattern offers considerable support for the null hypothesis: out of the 30 correlations reported in Table 3, 24 lend “moderate” support to the possibility that bilingualism does not affect cognitive performance.

**Table 3:** Correlation matrix for measures of bilingualism (BilVoc response times; BilVoc errors; and BilQ) and of attentional control (global measures in the ANT2:C&O and ANT2:A, as well as the conflict, orienting and alerting components from these tasks).

		Global Measures				Component Measures					

		ANT2:C&O		ANT1:A		Conflict		Orienting		Alerting	

		RT	Error	RT	Error	RT	Error	RT	Error	RT	Error

**Bilingualism**	*r*	.33	.20	.06	.24	.10	.06	.03	.21	.03	.12
**score for**	*p*	.002	.061	.675	.090	.341	.617	.809	.059	.822	.400
**BilVoc RTs**	*BF_01_*	0.07	1.32	**5.36**	1.44	**4.73**	**6.53**	**7.17**	1.28	**5.70**	**4.14**
	*BF_10_*	**14.08**	0.76	0.19	0.69	0.21	0.15	0.14	0.79	0.18	0.24
**Bilingualism**	*r*	–.03	–.02	.04	.04	–.05	–.07	–.03	–.02	–.10	–.07
**score for**	*p*	.781	.835	.789	.762	.651	.520	.799	.887	.492	.609
**BilVoc Errors**	*BF_01_*	**7.10**	**7.22**	**5.64**	**5.58**	**6.67**	**6.02**	**7.15**	**7.31**	**4.64**	**5.14**
	*BF_10_*	0.14	0.14	0.18	0.18	0.15	0.17	0.14	0.14	0.22	0.20
**Bilingualism**	*r*	–.12	–.06	.09	–.07	.12	.08	.20	–.07	–.16	.02
**score for**	*p*	.233	.567	.481	.588	.244	.471	.049	.486	.179	.867
**BilQ scores**	*BF01*	**3.85**	**6.60**	**5.21**	**5.76**	**3.98**	**6.00**	1.15	**6.11**	2.75	**6.56**
	*BF_10_*	0.26	0.15	0.19	0.17	0.25	0.17	0.87	0.16	0.36	0.15

*Note*: Bayes Factor (BF) values which suggest at least ‘moderate’ evidence (*BF* ≥ 3) are shown in bold.

### Measured possible confounding variables

SES and immigration data were collected but no discernible pattern of covariance with bilingualism was found (Table 2) so they were not entered as covariables in correlation analysis when testing for a bilingual advantage in attention. However, individualistic culture was found to negatively correlate with bilingualism as captured by BilVoc errors (*r* = –.28 *p* = .003, *BF_10_* = 9.4). This pattern is not surprising considering families with more individualist culture are often English in ancestry, and therefore the child can be expected to speak better English than Spanish. To further analyse this pattern, we repeated the analysis in which we probed for whether BilVoc errors could predict global or component measures, but Individualist culture was entered as a covariate. However, in this revised analysis again all results were not significant, *p* ≥ .664.

Additionally, we tested an a-priori hypothesis based on Tran, Arredondo and Yoshida’s (2015) argument that collectivist culture improves children’s receptiveness to additional information and therefore that their alerting system would be able to better take advantage of the double cue conditions in the ANT2 alerting trials (ANT2:A), resulting in a larger alerting effect. It would therefore stand to reason that individualist culture (the inverse of collectivism) would reduce one’s ability to take advantage of the double cue in the alerting condition. However, individualism had only a modest effect on the alerting effect RTs (*r* = .23, *p* = .07, *BF_10_* = 1.3) and actually produced an advantage in the alerting effect errors (*r* = .38, *p* = .002, *BF_10_* = 26.0). This indicates that for our sample, collectivist culture did not provide the alerting advantage hypothesised by Tran et al. (2015).

The full data and scripts used in analysis are available in open access on the Open Science Framework (https://osf.io/kc29b/).

## Discussion

In the current study, we explored the possibility that bilingualism conveys an advantage in ‘cognitive control’, and more specifically, in the ability to deploy attention. As outlined in the Introduction, it is very difficult if not impossible to match mono- and bilingual individuals on relevant variables other than linguality which might also affect cognitive control. Here, we recruited school children of varying degrees of bilingualism from the same population: the bilingual English/Spanish population of Gibraltar is the ideal natural control since they share largely the same education, amenities, and culture on a very small landmass. We captured the degree of bilingualism via both self-reported and ‘objective’ measures, and we measured cognitive control in an adaptation of existing tasks of auditory attention.

With 24 out of 30 correlations which probed the effect of bilingualism on cognitive control showing moderate evidence for the null hypothesis, this study has found overall no evidence that being bilingual affords attentional advantages in Gibraltarian children, and indeed has generated some weighted evidence that it does not. This is, of course, at odds with previous research reporting bilingual advantages for executive function (e.g., Green, 1988; Bialystok, 2001) as well as for the orienting and alerting systems (e.g., Tao et al., 2011; Anton et al., 2014; Tran, Arredondo & Yoshida, 2015; Yow & Li, 2015). However, it does support the growing literature of null findings regarding a bilingual advantage, such as recent results from the ABCD nationally representative cohort study (Dick, Garcia, Pruden et al., 2019). In this instance, it appears that a larger sample size, naturalistic control and measured confounder control has resulted in finding no advantage.

There was some evidence that the bilinguals in our sample may have performed worse. The substantial positive correlation between RTs in the BilVoc and the RTs in the ANT2 conflicting and orienting trials (ANT2:C&O) suggests that bilinguals performed worse overall in a test of attention, and contradicts the bilingual advantage hypothesis. While there is some speculation of negative consequences of bilingualism (e.g., Paap, Johnson & Sawi, 2015), this finding was not supported in our results by the other bilingual measures (BilVoc errors and BilQ) nor by global reaction time in the ANT2 alerting trials (ANT2:A) and so is likely either a random artefact, or the product of a highly complex, and not yet understood, mechanism. In the case of the latter, it is possible that the reason this appeared in two reaction time measures is because they are a complex measure which capture both sensory orientation and processing delays (Roebuck, Freigang & Barry, 2016). More insight into the exact stages of the cognitive processes involved in responding to these tests could be obtained using ‘mouse tracking’ methods which render more data about when in the stream of processing delays and errors occur (Damian, Ye, Oh & Zhang, 2018; Incera & McLennan, 2016).

### The natural control method to control random confounding effects

Using a natural control method, we have attempted to match bilinguals and monolinguals as closely as possible in a range of environmental, social, cultural factors which, if left uncontrolled, could bias the study. In this case this method has yielded evidence against a bilingual advantage in attention when monolinguals and bilinguals were closely matched. This supports Paap and colleagues’ (2013, 2015, 2016) claims that the ‘bilingual advantage’ reported in previous studies may be in part influenced by, or entirely caused by, confounding variables such as SES and immigrant status. However, to fully support this hypothesis the results of the natural control method must be replicated in different populations (e.g., Wales, Basque country, French Canada, many areas of Scandinavia and Northern Europe, or any small linguistic enclave similar to Gibraltar such as Italian/German speakers in Northern Italy).

We found evidence to support the observation that the factors associated with bilingualism can vary from population to population. We found that neither SES nor migrant status were associated with bilingual status, although culture was associated in one comparison. Previous studies have also failed to find a relationship between SES and cognitive ability (Paap, Johnson, Sawi & Greenberg, 2013) and bilingualism (Morton & Harper, 2007). We take this observation with our own data to make the suggestion that authors consider that confounding variables may act as random effects factors; that there exists no single list of confounding factors which will reliably bias studies. Random effects are unpredictable by nature which makes them difficult to control but the natural control method is uniquely placed to match bilinguals to monolinguals under these conditions. This is not to say that authors should abandon the identification and measurement of confounding factors, especially where an author has knowledge of the specific language and populations of study. Instead, we suggest the natural control as a complementary method which controls random confounding effects but comes with its own assumptions.

The standard approach in bilingual advantage research is to contrast bilinguals in one country with monolinguals from another (e.g., Antón et al., 2014; Duñabeitia, Hernández, Antón et al., 2014) under the assumption that differences between countries are controlled. This natural control method delivers a true control but makes two assumptions which have to be met: First, that by controlling the environment, monolinguals and bilinguals are matched. In the present study there is no evidence of SES and migrant status differences between bilinguals, and only one indication of cultural differences. Additionally the small and shared environment of Gibraltar leaves little room for unmeasured environmental differences so we conclude that this assumption is met. Second, that the shared environment does not change the nature of mono/bi-lingualism. In addition to the relative ease of accessing these populations, the standard approach has the merit of ensuring that the monolingual control is not affected by the bilingual group. In the present study, using a natural control, it is difficult to ensure that the monolingual controls were truly monolingual due to the presence of bilinguals and presence of both languages in their environment. Although it is not yet clear how these factors could influence a bilingual advantage, for caution’s sake the monolinguals in our control are perhaps best described instead as ‘not actively bilingual’, in that they do not speak two languages but are still the subjects of passive exposure. This is true for Gibraltar as much as any other massively bilingual region (e.g., linguistic enclaves) and so makes this a difficult assumption to meet. Therefore, the natural control method is not intended as a replacement for the standard approach but rather to complement it as both methods are necessary to investigate the bilingual advantage with respect to identifying confounding variables and ensuring true monolingual-bilingual comparisons.

### Linguistic specificity

Considering the phonological mechanisms which might underlie a bilingual advantage (e.g., phonological conflict: Spalek et al., 2014), it is possible that different languages and combinations of languages may exclusively produce/not produce bilingual advantages. There is growing evidence that languages with different phonologies produce different developmental (e.g., phonological awareness: Bialystok, Majumder & Martin, 2003), cognitive (e.g., switching costs: Prior & Gollan, 2011; Gollan et al., 2014; Declerck & Phillip, 2015; Phillip & Koch, 2016) and neurological (e.g., brain activation during articulation: Reverberi et al., 2015) effects.

It is also possible that the unique combination languages which arise when populations of two languages live together, known as creoles, should not produce a bilingual advantage because the vocabulary and syntax of both languages are combined into a single language. It is arguable therefore that no lexical conflict should arise when words from both languages are viewed as legal by the brain’s conflict monitoring processes. In Gibraltar a mix of the local creole, Llanito (Spanish:English ratio around 40:60), and pure English/Spanish are spoken. For this reason, Llanito was treat as a distinct language and not analogous to bilingualism in the BilQ. Comparison between Creole and ‘pure’ bilinguals could help elucidate language switching and lexical conflict effects.

### Assessing bilingualism using a behavioural questionnaire

Bilingualism has multiple components (e.g., vocabulary, spoken frequency, culture) and two measures were selected to capture a holistic measurement of bilingualism. An objective vocabulary size and retrieval speed test (BilVoc) was paired with a questionnaire (BilQ) to assess bilingual behaviours. Children were assessed instead of their parents in order to capture highly influential schooltime experiences (Burriss & Tsao, 2002) which the parents would not necessarily be privy to, though this was treat as a pilot measure since this may have introduced high measurement error (as with the BilVoc, ANT tests). The disagreement between some measures in this study is not surprising considering their different measurement domains and the background of poor convergent validity with attentional tests (Paap, Johnson & Sawi, 2015; 2016). However, future use of a child language behaviour questionnaire should be further supplemented by, and validated against, a parental questionnaire (e.g., Language and Social Background Questionnaire for the Bilingual Child: Redlinger, 1977).

### Studying the bilingual advantage in attention in children

Our study tested the attentional abilities of children of ages 9-10 our results, methodological considerations, and theory are most relevant for the developmental literature. However, the bilingual advantage has been found in children and shares the same theorised psycholinguistic mechanism (Barac & Bialystok, 2012; Bialystok, 1999; Bialystok & Martin, 2004; Bialystok, Martin, & Viswanathan, 2005; Carlson and Meltzoff, 2008; Kovacs & Mehler, 2009; Yang, Yang & Lust, 2011). It has been argued that the attentional advantages of a bilingual advantage would be more pronounced in young children and older adults because they are not operating at ceiling on measures of cognitive control, and thus an advantage over one’s peers would be more measurable (e.g., Bialystok, 2017).

Relatively few studies have investigated a bilingual advantage in attention in children and have gleaned similar results using similar methods to the present study. In a study using the same attentional task to the present study, Antón and colleagues (2014) matched Spanish monolingual and Basque–Spanish bilingual children and found no significant difference in the conflicting, alerting nor global components of an ANT flanker task. Duñabeitia and colleagues (2014) tested Spanish monolingual and Basque–Spanish bilingual children across a range of ages across 6 grades and found no significant difference on verbal Stroop and number-size congruency tasks at any age. Antón, Carreiras, and Duñabeitia (2019) matched bilinguals and monolinguals and found no significant advantage across a range of attention tasks (Flanker, Simon, and Stroop variants) in neither interference nor global scores. However, these predominantly Basque (Basque Center for Cognition, Brain, and Language) studies, in common with our study, study relatively prosperous linguistic enclaves located on the Iberian peninsula which speak Spanish and so are not necessarily representative of other areas.

### Summary

In our study, no evidence of a bilingual advantage in attention was found when bilinguals and monolinguals were matched on a variety of factors and situated in a natural control. Geo-political factors appear to play a role as part of complex dynamical systems, and while their exact involvement is not yet clear, previous studies suggest that they confound the bilingual advantage. We find evidence that this is true; factors which have previously been identified as playing a confounding role failed to do so. The natural control may be a useful addition to the bilingual advantage researchers’ toolkit for controlling random confounding effects. This method is constrained by two main assumptions and these results may be specific to the languages, bilingual culture, and ages studied.

## Data Accessibility Statement

The full data, materials, and scripts used in analysis are available in open access on the Open Science Framework (https://osf.io/kc29b/).

## Additional File

The additional file for this article can be found as follows:

10.5334/joc.94.s1 Appendix S1. Example scoring the language usage questionnaire for English language usage.
