# Supplementary material for: Employing Natural Control for Confounding Factors in the Hunt for the Bilingual Advantage in Attention: Evidence from School Children in Gibraltar
Source: J Cogn. 2020 Mar 20;3(1):5. doi: 10.5334/joc.94 (PMC7082826; doi:10.5334/joc.94)
Supplement: Appendix S1. — Example scoring the language usage questionnaire for English language usage. [file joc-3-1-94-s1.pdf]

Text summary: Appendix A includes information on how the language usage questionnaire was scored, using an example for scoring English language usage.

Appendix S1

Example scoring the language usage questionnaire for English language usage

**Questions used**	**Response**	**Score**
**1e**	Never<br>A few times a month<br>Every day<br>Always	**1**<br>**2**<br>**3**<br>**4**
**2e**	Never<br>A few times a month<br>Every day<br>Always	**1**<br>**2**<br>**3**<br>**4**
**3e**	At school<br>Years before school<br>I’ve always known it	**1**<br>**2**<br>**3**
**4**	English<br>Spanish<br>Both	**2**<br>**0**<br>**1**
**5**	English<br>Spanish<br>Both	**2**<br>**0**<br>**1**
**6**	English<br>Spanish<br>Both	**2**<br>**0**

		**1**

Note: Question numbers correspond to questions displayed in Table 1. For Spanish language, subquestions 1s, 2s, & 3s would be used, and scoring would be flipped for questions 4, 5 & 6.
